# 4-Chloro-*N*-(3,4-dimethyl­phen­yl)-2-methyl­benzene­sulfonamide

**DOI:** 10.1107/S1600536811041341

**Published:** 2011-10-12

**Authors:** Vinola Z. Rodrigues, Sabine Foro, B. Thimme Gowda

**Affiliations:** a Department of Chemistry, Mangalore University, Mangalagangotri 574 199, Mangalore, India; b Institute of Materials Science, Darmstadt University of Technology, Petersenstrasse 23, D-64287 Darmstadt, Germany

## Abstract

In the title compound, C_15_H_16_ClNO_2_S, the conformation of the N—C bond in the C—SO_2_—NH—C segment is *gauche* with respect to the S=O bonds. Further, the N—H bond in the C—SO_2_—NH—C segment is *syn* with respect to the *meta*-methyl group in the aniline benzene ring and the *ortho*-methyl group in the sulfonyl benzene ring. The C—SO_2_—NH—C torsion angle is −49.72 (18)°. The sulfonyl and aniline benzene rings are tilted relative to each other by 71.6 (1)°. The crystal structure features inversion-related dimers linked by pairs of N—H⋯O hydrogen bonds.

## Related literature

For the preparation of the title compound, see: Savitha & Gowda (2006 ▶). For hydrogen-bonding modes of sulfonamides, see: Adsmond & Grant (2001 ▶). For studies on the effects of substituents on the structures and other aspects of *N*-(ar­yl)-amides, see: Gowda *et al.* (2007*a* ▶), on *N*-(ar­yl)-methane­sulfonamides, see: Gowda *et al.* (2007*b* ▶), on *N*-(ar­yl)-aryl­sulfonamides, see: Gelbrich *et al.* (2007 ▶); Perlovich *et al.* (2006 ▶); Rodrigues *et al.* (2011 ▶); Shetty & Gowda (2005 ▶) and on *N*-(chloro)-aryl­sulfonamides, see: Gowda *et al.* (2003 ▶).
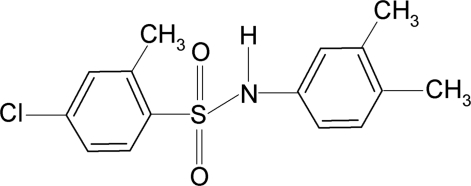


## Experimental

### 

#### Crystal data

C_15_H_16_ClNO_2_S
*M* *_r_* = 309.80
Triclinic, 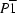

*a* = 8.1321 (9) Å
*b* = 8.1604 (9) Å
*c* = 12.456 (1) Å
α = 80.991 (9)°
β = 72.903 (8)°
γ = 78.979 (8)°
*V* = 771.04 (14) Å^3^
*Z* = 2
Mo *K*α radiation
μ = 0.38 mm^−1^
*T* = 293 K
0.50 × 0.44 × 0.40 mm

#### Data collection

Oxford Diffraction Xcalibur diffractometer with a Sapphire CCD detector
Absorption correction: multi-scan (*CrysAlis RED*; Oxford Diffraction, 2009 ▶) *T*_min_ = 0.832, *T*_max_ = 0.862
5220 measured reflections
3126 independent reflections
2631 reflections with *I* > 2σ(*I*)
*R*_int_ = 0.008

#### Refinement

*R*[*F* ^2^ > 2σ(*F* ^2^)] = 0.037
*wR*(*F* ^2^) = 0.109
*S* = 1.05
3126 reflections
187 parameters
1 restraint
H atoms treated by a mixture of independent and constrained refinement
Δρ_max_ = 0.25 e Å^−3^
Δρ_min_ = −0.28 e Å^−3^

### 

Data collection: *CrysAlis CCD* (Oxford Diffraction, 2009 ▶); cell refinement: *CrysAlis RED* (Oxford Diffraction, 2009 ▶); data reduction: *CrysAlis RED*; program(s) used to solve structure: *SHELXS97* (Sheldrick, 2008 ▶); program(s) used to refine structure: *SHELXL97* (Sheldrick, 2008 ▶); molecular graphics: *PLATON* (Spek, 2009 ▶); software used to prepare material for publication: *SHELXL97*.

## Supplementary Material

Crystal structure: contains datablock(s) I, global. DOI: 10.1107/S1600536811041341/nc2250sup1.cif

Structure factors: contains datablock(s) I. DOI: 10.1107/S1600536811041341/nc2250Isup2.hkl

Supplementary material file. DOI: 10.1107/S1600536811041341/nc2250Isup3.cml

Additional supplementary materials:  crystallographic information; 3D view; checkCIF report

## Figures and Tables

**Table 1:** Hydrogen-bond geometry (Å, °)

*D*—H⋯*A*	*D*—H	H⋯*A*	*D*⋯*A*	*D*—H⋯*A*
N1—H1*N*⋯O1^i^	0.83 (2)	2.07 (2)	2.899 (2)	175 (2)

Symmetry code: (i) 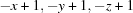.

**Fig. 1.**
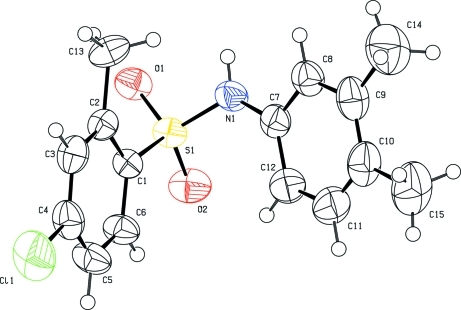
Molecular structure of the title compound, showing the atom labelling scheme and displacement ellipsoids are drawn at the 50% probability level.

**Fig. 2.**
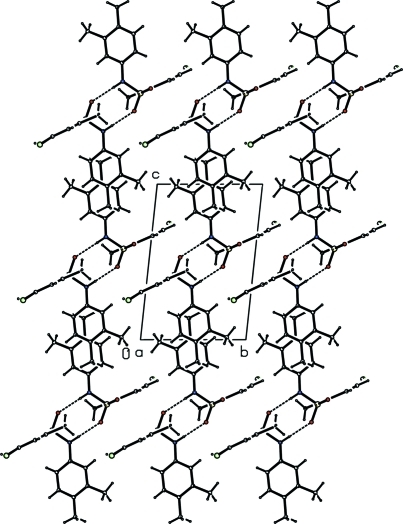
Molecular packing of (I) with hydrogen bonding shown as dashed lines.

## supplementary crystallographic information

### Comment

The sulfonamide moiety is the constituent of many biologically important compounds. The hydrogen bonding preferences of sulfonamides have been investigated (Adsmond & Grant, 2001). As part of our studies on the substituent effects on the structures and other aspects of *N*-(aryl)-amides (Gowda *et al.*, 2007*a*), *N*-(aryl)-methanesulfonamides (Gowda *et al.*, 2007*b*), *N*-(aryl)-arylsulfonamides (Rodrigues *et al.*, 2011; Shetty & Gowda, 2005) and *N*-(chloro)-arylsulfonamides (Gowda *et al.*, 2003), in the present work, the crystal structure of 4-Chloro-2-methyl-*N*-(3,4-dimethylphenyl)benzenesulfonamide (I) has been determined (Fig. 1).

In (I), the N—H bond in the C—SO_2_—NH—C segment is *syn* with respect to the *meta*-methyl group in the anilino benzene ring and *ortho*-methyl group in the sulfonyl benzene ring. The molecule is bent at the S atom with the C—SO_2_—NH—C torsion angle of -49.7 (2), compared to the value of 67.5 (2) in 4-Chloro-2-methyl-*N*- (2,4-dimethylphenyl)benzenesulfonamide (II) (Rodrigues *et al.*, 2011).

The sulfonyl and the aniline benzene rings are tilted relative to each other by 71.6 (1)°, compared to the values of 44.5 (1)° in (II).

The other bond parameters in (I) are similar to those observed in (II) and other aryl sulfonamides (Perlovich *et al.*, 2006; Gelbrich *et al.*, 2007).

In the crystal structure two molecules each are linked by intermolecular N–H···O hydrogen bonding into dimers that are located on centres of inversion (Fig. 2).

### Experimental

The solution of *m*-chlorotoluene (10 ml) in chloroform (40 ml) was treated dropwise with chlorosulfonic acid (25 ml) at 0 ° C. After the initial evolution of hydrogen chloride subsided, the reaction mixture was brought to room temperature and poured into crushed ice in a beaker. The chloroform layer was separated, washed with cold water and allowed to evaporate slowly. The residual 2-methyl-4-chlorobenzenesulfonylchloride was treated with 3,4-dimethylaniline in the stoichiometric ratio and boiled for ten minutes. The reaction mixture was then cooled to room temperature and added to ice cold water (100 ml). The resultant solid 4-chloro-2-methyl-*N*- (3,4-dimethylphenyl)-benzenesulfonamide was filtered under suction and washed thoroughly with cold water. It was then recrystallized to constant melting point from dilute ethanol. The purity of the compound was checked and characterized by recording its infrared and NMR spectra (Savitha & Gowda, 2006).

Prism like colourless single crystals used in X-ray diffraction studies were grown in ethanolic solution by slow evaporation of the solvent at room temperature.

### Refinement

The H atoms of the NH groups were located in a difference map and later restrained to N—H = 0.86 (2) %A. The other H atoms were positioned with idealized geometry using a riding model with the aromatic C—H = 0.93Å and methyl C—H = 0.96 Å. All H atoms were refined with isotropic displacement parameters. The *U*_iso_(H) values were set at 1.2*U*_eq_(C-aromatic, N) and 1.5*U*_eq_(*C*-methyl).

### Crystal data

**Table:**

C_15_H_16_ClNO_2_S	*Z* = 2
*M**_r_* = 309.80	*F*(000) = 324
Triclinic, *P*1	*D*_x_ = 1.334 Mg m^−^^3^
Hall symbol: -P 1	Mo *K*α radiation, λ = 0.71073 Å
*a* = 8.1321 (9) Å	Cell parameters from 3090 reflections
*b* = 8.1604 (9) Å	θ = 2.6–27.8°
*c* = 12.456 (1) Å	µ = 0.38 mm^−^^1^
α = 80.991 (9)°	*T* = 293 K
β = 72.903 (8)°	Prism, colourless
γ = 78.979 (8)°	0.50 × 0.44 × 0.40 mm
*V* = 771.04 (14) Å^3^	

### Data collection

**Table:**

Oxford Diffraction Xcalibur diffractometer with a Sapphire CCD detector	3126 independent reflections
Radiation source: fine-focus sealed tube	2631 reflections with *I* > 2σ(*I*)
graphite	*R*_int_ = 0.008
Rotation method data acquisition using ω scans	θ_max_ = 26.4°, θ_min_ = 2.7°
Absorption correction: multi-scan (*CrysAlis RED*; Oxford Diffraction, 2009)	*h* = −9→10
*T*_min_ = 0.832, *T*_max_ = 0.862	*k* = −8→10
5220 measured reflections	*l* = −14→15

### Refinement

**Table:**

Refinement on *F*^2^	Primary atom site location: structure-invariant direct methods
Least-squares matrix: full	Secondary atom site location: difference Fourier map
*R*[*F*^2^ > 2σ(*F*^2^)] = 0.037	Hydrogen site location: inferred from neighbouring sites
*wR*(*F*^2^) = 0.109	H atoms treated by a mixture of independent and constrained refinement
*S* = 1.05	*w* = 1/[σ^2^(*F*_o_^2^) + (0.0545*P*)^2^ + 0.2665*P*] where *P* = (*F*_o_^2^ + 2*F*_c_^2^)/3
3126 reflections	(Δ/σ)_max_ < 0.001
187 parameters	Δρ_max_ = 0.25 e Å^−^^3^
1 restraint	Δρ_min_ = −0.28 e Å^−^^3^

### Special details

**Table:**

Geometry. All e.s.d.'s (except the e.s.d. in the dihedral angle between two l.s. planes) are estimated using the full covariance matrix. The cell e.s.d.'s are taken into account individually in the estimation of e.s.d.'s in distances, angles and torsion angles; correlations between e.s.d.'s in cell parameters are only used when they are defined by crystal symmetry. An approximate (isotropic) treatment of cell e.s.d.'s is used for estimating e.s.d.'s involving l.s. planes.
Refinement. Refinement of *F*^2^ against ALL reflections. The weighted *R*-factor *wR* and goodness of fit *S* are based on *F*^2^, conventional *R*-factors *R* are based on *F*, with *F* set to zero for negative *F*^2^. The threshold expression of *F*^2^ > σ(*F*^2^) is used only for calculating *R*-factors(gt) *etc*. and is not relevant to the choice of reflections for refinement. *R*-factors based on *F*^2^ are statistically about twice as large as those based on *F*, and *R*- factors based on ALL data will be even larger.

### Fractional atomic coordinates and isotropic or equivalent isotropic displacement parameters (Å^2^)

**Table:**

	*x*	*y*	*z*	*U*_iso_*/*U*_eq_	
C1	0.2471 (2)	0.8601 (2)	0.62239 (14)	0.0382 (4)	
C2	0.1044 (2)	0.8022 (2)	0.60861 (15)	0.0425 (4)	
C3	−0.0582 (2)	0.8982 (3)	0.64517 (17)	0.0523 (5)	
H3	−0.1560	0.8638	0.6368	0.063*	
C4	−0.0766 (3)	1.0436 (3)	0.69355 (18)	0.0562 (5)	
C5	0.0646 (3)	1.1022 (3)	0.7051 (2)	0.0609 (5)	
H5	0.0506	1.2017	0.7366	0.073*	
C6	0.2272 (3)	1.0094 (2)	0.66863 (18)	0.0508 (4)	
H6	0.3245	1.0470	0.6751	0.061*	
C7	0.3843 (2)	0.5413 (2)	0.77995 (16)	0.0470 (4)	
C8	0.3257 (3)	0.3903 (3)	0.82440 (17)	0.0530 (5)	
H8	0.3325	0.3120	0.7759	0.064*	
C9	0.2568 (3)	0.3528 (3)	0.93980 (19)	0.0638 (6)	
C10	0.2442 (3)	0.4724 (3)	1.01236 (19)	0.0698 (6)	
C11	0.3023 (4)	0.6217 (3)	0.9667 (2)	0.0740 (7)	
H11	0.2931	0.7014	1.0148	0.089*	
C12	0.3742 (3)	0.6588 (3)	0.85186 (19)	0.0639 (6)	
H12	0.4148	0.7604	0.8236	0.077*	
C13	0.1185 (3)	0.6361 (3)	0.55858 (18)	0.0578 (5)	
H13A	0.1527	0.5428	0.6089	0.069*	
H13B	0.2039	0.6358	0.4865	0.069*	
H13C	0.0077	0.6269	0.5493	0.069*	
C14	0.1992 (5)	0.1838 (4)	0.9841 (3)	0.1010 (10)	
H14A	0.2671	0.1238	1.0333	0.121*	
H14B	0.2158	0.1205	0.9219	0.121*	
H14C	0.0783	0.1995	1.0251	0.121*	
C15	0.1678 (4)	0.4386 (5)	1.1389 (2)	0.0994 (10)	
H15A	0.2363	0.3421	1.1679	0.119*	
H15B	0.0503	0.4178	1.1543	0.119*	
H15C	0.1683	0.5344	1.1747	0.119*	
N1	0.4560 (2)	0.5674 (2)	0.66087 (13)	0.0489 (4)	
H1N	0.459 (3)	0.490 (2)	0.6238 (17)	0.059*	
O1	0.50734 (18)	0.70770 (17)	0.47026 (11)	0.0528 (3)	
O2	0.56945 (17)	0.83991 (19)	0.61489 (13)	0.0601 (4)	
Cl1	−0.28370 (8)	1.15422 (10)	0.74513 (7)	0.0927 (3)	
S1	0.46083 (5)	0.74755 (5)	0.58477 (4)	0.04260 (15)	

### Atomic displacement parameters (Å^2^)

**Table:**

	*U*^11^	*U*^22^	*U*^33^	*U*^12^	*U*^13^	*U*^23^
C1	0.0340 (8)	0.0368 (8)	0.0425 (9)	−0.0066 (6)	−0.0080 (7)	−0.0035 (7)
C2	0.0397 (9)	0.0476 (10)	0.0416 (9)	−0.0115 (7)	−0.0112 (7)	−0.0030 (7)
C3	0.0374 (9)	0.0666 (12)	0.0546 (11)	−0.0082 (8)	−0.0173 (8)	−0.0018 (9)
C4	0.0414 (10)	0.0598 (12)	0.0581 (12)	0.0088 (9)	−0.0117 (9)	−0.0028 (10)
C5	0.0567 (12)	0.0448 (11)	0.0783 (15)	0.0036 (9)	−0.0152 (10)	−0.0177 (10)
C6	0.0441 (10)	0.0429 (10)	0.0679 (12)	−0.0083 (8)	−0.0148 (9)	−0.0123 (9)
C7	0.0381 (9)	0.0514 (10)	0.0469 (10)	0.0059 (8)	−0.0108 (7)	−0.0084 (8)
C8	0.0468 (10)	0.0525 (11)	0.0541 (11)	0.0020 (8)	−0.0115 (8)	−0.0057 (9)
C9	0.0506 (12)	0.0708 (14)	0.0592 (13)	0.0004 (10)	−0.0117 (10)	0.0069 (11)
C10	0.0596 (13)	0.0875 (17)	0.0487 (12)	0.0105 (12)	−0.0100 (10)	−0.0033 (12)
C11	0.0895 (18)	0.0761 (16)	0.0506 (12)	0.0070 (13)	−0.0164 (12)	−0.0185 (11)
C12	0.0746 (14)	0.0592 (12)	0.0561 (12)	−0.0013 (11)	−0.0179 (11)	−0.0114 (10)
C13	0.0491 (11)	0.0725 (13)	0.0640 (12)	−0.0269 (10)	−0.0165 (9)	−0.0197 (10)
C14	0.115 (3)	0.095 (2)	0.082 (2)	−0.0341 (19)	−0.0141 (18)	0.0186 (17)
C15	0.097 (2)	0.125 (3)	0.0514 (14)	0.0077 (19)	−0.0032 (14)	−0.0002 (15)
N1	0.0541 (9)	0.0423 (8)	0.0455 (9)	0.0010 (7)	−0.0086 (7)	−0.0105 (7)
O1	0.0552 (8)	0.0490 (7)	0.0465 (7)	−0.0105 (6)	0.0014 (6)	−0.0087 (6)
O2	0.0358 (7)	0.0655 (9)	0.0825 (10)	−0.0134 (6)	−0.0112 (6)	−0.0214 (8)
Cl1	0.0543 (4)	0.1043 (5)	0.1042 (5)	0.0281 (3)	−0.0195 (3)	−0.0198 (4)
S1	0.0331 (2)	0.0432 (2)	0.0493 (3)	−0.00675 (17)	−0.00475 (17)	−0.00988 (18)

### Geometric parameters (Å, °)

**Table:**

C1—C6	1.390 (2)	C10—C11	1.369 (4)
C1—C2	1.397 (2)	C10—C15	1.517 (3)
C1—S1	1.7692 (17)	C11—C12	1.386 (3)
C2—C3	1.390 (3)	C11—H11	0.9300
C2—C13	1.549 (3)	C12—H12	0.9300
C3—C4	1.377 (3)	C13—H13A	0.9600
C3—H3	0.9300	C13—H13B	0.9600
C4—C5	1.377 (3)	C13—H13C	0.9600
C4—Cl1	1.740 (2)	C14—H14A	0.9600
C5—C6	1.378 (3)	C14—H14B	0.9600
C5—H5	0.9300	C14—H14C	0.9600
C6—H6	0.9300	C15—H15A	0.9600
C7—C8	1.381 (3)	C15—H15B	0.9600
C7—C12	1.387 (3)	C15—H15C	0.9600
C7—N1	1.423 (2)	N1—S1	1.6202 (17)
C8—C9	1.389 (3)	N1—H1N	0.832 (16)
C8—H8	0.9300	O1—S1	1.4368 (14)
C9—C10	1.401 (4)	O2—S1	1.4237 (14)
C9—C14	1.511 (4)		
			
C6—C1—C2	121.21 (16)	C12—C11—H11	118.6
C6—C1—S1	116.39 (13)	C11—C12—C7	118.6 (2)
C2—C1—S1	122.39 (13)	C11—C12—H12	120.7
C3—C2—C1	117.21 (17)	C7—C12—H12	120.7
C3—C2—C13	119.28 (16)	C2—C13—H13A	109.5
C1—C2—C13	123.48 (16)	C2—C13—H13B	109.5
C4—C3—C2	120.94 (18)	H13A—C13—H13B	109.5
C4—C3—H3	119.5	C2—C13—H13C	109.5
C2—C3—H3	119.5	H13A—C13—H13C	109.5
C3—C4—C5	121.76 (18)	H13B—C13—H13C	109.5
C3—C4—Cl1	119.51 (16)	C9—C14—H14A	109.5
C5—C4—Cl1	118.70 (17)	C9—C14—H14B	109.5
C4—C5—C6	118.19 (19)	H14A—C14—H14B	109.5
C4—C5—H5	120.9	C9—C14—H14C	109.5
C6—C5—H5	120.9	H14A—C14—H14C	109.5
C5—C6—C1	120.65 (18)	H14B—C14—H14C	109.5
C5—C6—H6	119.7	C10—C15—H15A	109.5
C1—C6—H6	119.7	C10—C15—H15B	109.5
C8—C7—C12	119.48 (19)	H15A—C15—H15B	109.5
C8—C7—N1	117.62 (18)	C10—C15—H15C	109.5
C12—C7—N1	122.89 (19)	H15A—C15—H15C	109.5
C7—C8—C9	121.6 (2)	H15B—C15—H15C	109.5
C7—C8—H8	119.2	C7—N1—S1	125.99 (13)
C9—C8—H8	119.2	C7—N1—H1N	117.0 (16)
C8—C9—C10	119.0 (2)	S1—N1—H1N	112.4 (16)
C8—C9—C14	119.3 (2)	O2—S1—O1	118.08 (9)
C10—C9—C14	121.7 (2)	O2—S1—N1	109.61 (9)
C11—C10—C9	118.6 (2)	O1—S1—N1	104.70 (8)
C11—C10—C15	120.3 (3)	O2—S1—C1	106.69 (8)
C9—C10—C15	121.1 (3)	O1—S1—C1	110.69 (8)
C10—C11—C12	122.7 (2)	N1—S1—C1	106.56 (8)
C10—C11—H11	118.6		
			
C6—C1—C2—C3	−1.3 (3)	C8—C9—C10—C15	179.2 (2)
S1—C1—C2—C3	177.61 (13)	C14—C9—C10—C15	−1.5 (4)
C6—C1—C2—C13	−179.45 (18)	C9—C10—C11—C12	−0.5 (4)
S1—C1—C2—C13	−0.6 (3)	C15—C10—C11—C12	179.6 (2)
C1—C2—C3—C4	−0.4 (3)	C10—C11—C12—C7	1.4 (4)
C13—C2—C3—C4	177.81 (19)	C8—C7—C12—C11	−1.1 (3)
C2—C3—C4—C5	1.7 (3)	N1—C7—C12—C11	−179.9 (2)
C2—C3—C4—Cl1	−176.56 (14)	C8—C7—N1—S1	153.78 (15)
C3—C4—C5—C6	−1.3 (3)	C12—C7—N1—S1	−27.4 (3)
Cl1—C4—C5—C6	177.06 (16)	C7—N1—S1—O2	65.36 (18)
C4—C5—C6—C1	−0.5 (3)	C7—N1—S1—O1	−167.05 (16)
C2—C1—C6—C5	1.8 (3)	C7—N1—S1—C1	−49.72 (18)
S1—C1—C6—C5	−177.18 (16)	C6—C1—S1—O2	1.39 (17)
C12—C7—C8—C9	−0.1 (3)	C2—C1—S1—O2	−177.56 (14)
N1—C7—C8—C9	178.80 (17)	C6—C1—S1—O1	−128.29 (15)
C7—C8—C9—C10	1.0 (3)	C2—C1—S1—O1	52.77 (16)
C7—C8—C9—C14	−178.3 (2)	C6—C1—S1—N1	118.43 (15)
C8—C9—C10—C11	−0.7 (3)	C2—C1—S1—N1	−60.51 (16)
C14—C9—C10—C11	178.6 (2)		

### Hydrogen-bond geometry (Å, °)

**Table:**

*D*—H···*A*	*D*—H	H···*A*	*D*···*A*	*D*—H···*A*
N1—H1N···O1^i^	0.83 (2)	2.07 (2)	2.899 (2)	175 (2)

Symmetry codes: (i) −*x*+1, −*y*+1, −*z*+1.

## References

[bb1] Adsmond, D. A. & Grant, D. J. W. (2001). *J. Pharm. Sci.* **90**, 2058–2077. 10.1002/jps.1157 11745765

[bb2] Gelbrich, T., Hursthouse, M. B. & Threlfall, T. L. (2007). *Acta Cryst.* B**63**, 621–632. 10.1107/S010876810701395X 17641433

[bb3] Gowda, B. T., Foro, S. & Fuess, H. (2007*a*). *Acta Cryst.* E**63**, o1975–o1976.

[bb4] Gowda, B. T., Foro, S. & Fuess, H. (2007*b*). *Acta Cryst.* E**63**, o2339.

[bb5] Gowda, B. T. & Kumar, B. H. A. (2003). *Oxid. Commun. A*, **26**, 403–425.

[bb6] Oxford Diffraction (2009). *CrysAlis CCD* and *CrysAlis RED* Oxford Diffraction Ltd, Yarnton, England.

[bb7] Perlovich, G. L., Tkachev, V. V., Schaper, K.-J. & Raevsky, O. A. (2006). *Acta Cryst.* E**62**, o780–o782.

[bb8] Rodrigues, V. Z., Foro, S. & Gowda, B. T. (2011). *Acta Cryst.* E**67**, o2648. 10.1107/S1600536811036956 PMC3201300 22058774

[bb9] Savitha, M. B. & Gowda, B. T. (2006). *Z. Naturforsch. Teil A*, **61**, 600–606.

[bb10] Sheldrick, G. M. (2008). *Acta Cryst.* A**64**, 112–122. 10.1107/S0108767307043930 18156677

[bb11] Shetty, M. & Gowda, B. T. (2005). *Z. Naturforsch. Teil A*, **60**, 113–120.

[bb12] Spek, A. L. (2009). *Acta Cryst.* D**65**, 148–155. 10.1107/S090744490804362X PMC2631630 19171970

